# Modifications to the Active Living Every Day (ALED) Course for Adults With Arthritis

**Published:** 2007-06-15

**Authors:** Leigh F Callahan, Britta Schoster, Katherine Buysse, Jennifer Hootman, Teresa Brady, Laura Sally, Katrina Donahue, Thelma Mielenz

**Affiliations:** University of North Carolina at Chapel Hill; Thurston Arthritis Research Center, Chapel Hill, NC; Thurston Arthritis Research Center, Chapel Hill, NC; Centers for Disease Control and Prevention, Atlanta, Ga; Centers for Disease Control and Prevention, Atlanta, Ga; School of Public Health, Department of Health Behavior and Health Education, University of North Carolina-Chapel Hill, Chapel Hill, NC; Department of Family Medicine, University of North Carolina-Chapel Hill, Chapel Hill, NC; Thurston Arthritis Research Center and Department of Physical Therapy, University of North Carolina-Chapel Hill, Chapel Hill, NC

## Abstract

**Introduction:**

Active Living Every Day (ALED) is a 20-week behavioral theory-based physical activity program originally developed for the general population; the purpose of our qualitative evaluation was to investigate whether the existing program is also appropriate (regarding safety, content, and instructor training) for sedentary adults with arthritis.

**Methods:**

We conducted telephone interviews with 30 of 355 participants in a randomized control trial of the ALED program for sedentary adults with arthritis within 6 months after they completed the program. Interviewees, who attended at least 50% of program classes, were asked about the safety of program activities, the knowledge they gained from the program, how they felt about their program instructors, and any recommendations they had for how the program could be modified to better serve people with arthritis. We used NUD*IST (N6) (QSR International, Melbourne, Australia) software for the qualitative data analysis. We also conducted a conference call with program instructors to elicit their opinion of the course and how it might be improved to better meet the needs of people with arthritis.

**Results:**

Twenty seven (90%) of the program participants we interviewed were female, and their average age was 69 years. Components of the course that they reported finding particularly helpful were being encouraged to exercise “bit by bit” and receiving social support from other adults with arthritis. Program instructors and program participants both generally felt that the program was appropriate for people with arthritis but could be enhanced with the following modifications: 1) incorporating arthritis-specific information in the textbook, 2) providing information on pain management, 3) and providing dietary and nutritional information for arthritis management. Instructors also reported a need for more information on pain management and arthritis during their training.

**Conclusion:**

Although instructors and participants felt the ALED program as designed for the general population was useful for people with arthritis, they suggested minor modifications that would make the program even more beneficial. Some of these modifications may be applicable to other community-based activity programs not designed specifically for people with arthritis.

## Introduction

Arthritis is an important public health problem that causes a significant burden on our society (1). It is the most prevalent chronic condition and the leading cause of disability in the United States (2). Numerous studies have shown that engaging in physical activity is beneficial for people with arthritis (3-12). However, although physical activity programs have been developed specifically for people with arthritis (13), less than 1% of Americans with arthritis have enrolled in or participated in these programs (14,15). Increasing the number of physical activity programs offered has the potential to increase the rate of participation among people with arthritis. A number of programs developed for more general audiences could possibly be adapted for use in a target population of people with arthritis.

One such program, Active Living Every Day (ALED), is a behavioral theory-based physical activity program designed to teach people the cognitive and behavioral skills necessary to become and stay physically active (16-19). Developed jointly by the Cooper Institute, Brown University, and Human Kinetics, ALED is a 20-lesson course in which participants meet once a week for an hour-long group session structured around the ALED textbook (20) during which they discuss ways to identify and overcome barriers to physical activity. There is no exercise in the ALED class itself. All participants receive the ALED textbook and a pedometer to be used outside of class to monitor the number of steps they take and thus motivate themselves to be more active. The textbook reviews the main points covered in the classes (i.e., setting goals, enlisting support, and managing time) and also contains worksheets and assignments. If participants have access to the Internet and choose to be independent learners, they can take the course online rather than attend the classes. One study of ALED showed that it can be as effective as a structured exercise program in increasing participants' level of physical activity, improving their cardiorespiratory fitness, and reducing their blood pressure (19). Another showed that the program can be successfully translated into community settings with diverse populations (21).

ALED was developed for the general population, and previous studies of the program excluded people with arthritis (16-19). The two main purposes of our qualitative evaluation were 1) to investigate whether the existing ALED program is also appropriate for sedentary adults with arthritis in terms of safety, course content, and instructor training and support and 2) to develop, if necessary, a set of recommendations for modifying the existing ALED curriculum to better meet the needs of people with arthritis.

## Methods

### General ALED study design   

In February 2004, we began a 20-week randomized controlled trial (RCT) of ALED among 355 adults with arthritis residing in 17 urban or rural community settings across North Carolina. Potential participants were screened by the ALED project managers by phone. Eligibility requirements for participating in the RCT were being aged 18 years or older, exercising fewer than three times per week, and having reported doctor-diagnosed arthritis in responses on the Behavioral Risk Factor Surveillance System (BRFSS) 2002 arthritis and physical activity survey modules (22). The three most common conditions reported by participants in the RCT were osteoarthritis, fibromyalgia, and rheumatoid arthritis, which are also the most prevalent types of arthritis in the United States according to the Arthritis Foundation (1). RCT participants completed baseline and follow-up self-report questionnaires assessing their functional and psychological health status; they also underwent physical tests to measure their physical functioning and fitness. We used two measures assessed in the RCT to stratify the responses of participants in this qualitative study: 1) participants’ difficulty in performing activities of daily living (ADLs) as measured by the Health Assessment Questionnaire Disability Index (HAQ-DI), with possible scores ranging from 0 (can perform an activity with no difficulty) to 3 (unable to perform the activity) (23), and 2) participants’ self-reported level of pain as measured by the visual analog scale (VAS), with possible scores ranging from 0 (no pain) to 100 (pain as bad as it could be) based on the multidimensional HAQ (24).

Participants in the RCT were randomly assigned to either an intervention group or a control (delayed-intervention) group. The intervention group received the course upon enrollment in the study, and the control group was offered the ALED course after the trial was over. We did not follow the control group after the intervention group completed the ALED course. Members of the control group completed baseline and final assessment questionnaires with members of the intervention group.

All instructors (n = 20) were trained by a master trainer according to a standardized ALED protocol, and 17 ALED classes were initiated statewide. Most classes were conducted in senior centers, community health centers, and hospital wellness centers. Each class followed the standardized ALED format, had an average of 10 participants per class, and met once per week for 20 weeks. Instructors were recruited through the North Carolina Area Agencies on Aging; all had a history of some type of community-based health work, but they did not necessarily have experience working with people with arthritis. The instructor recruitment was designed to select people representative of those working with state health agencies in community health settings. Instructors received teaching materials and a CD-ROM, completed an online test, and received certification from Human Kinetics, distributors of the course, prior to beginning their classes. In response to an instructor's inquiry about general arthritis information, the ALED project managers provided all instructors with free pamphlets from the Arthritis Foundation and the National Institute of Arthritis and Musculoskeletal Skin Diseases covering a variety of arthritis-related topics (e.g., arthritis and fatigue, back pain, hip replacement).

In addition to the ALED training, instructors received information about the study, and all completed National Institutes of Health human subjects training. They were asked to query participants at the beginning of each class about any adverse health events and were told to report any such events immediately to the research team. The research team established a toll-free telephone number through which the instructors could contact the project directors and the study's principal investigator, as well as a listserv to facilitate communication among the instructors. The primary quantitative results from the study will be reported elsewhere. All protocols for the study were approved by the Institutional Review Board of the University of North Carolina School of Medicine.

### Qualitative ALED study design 

#### Participant sample

For the purpose of the qualitative evaluation reported here, we first classified the 355 RCT participants (in either the intervention or control group), as "completers" or "noncompleters," defining "completers" as those who attended at least 50% of the classes and "noncompleters" as those who attended less than 50%. We sampled only the completers, because they had more exposure to the course and thus were likely to be better able to comment on their experience. We randomly chose two completers from each ALED community site (one from the intervention group and one from the control [delayed-intervention] group) to participate in a phone interview, with a goal of conducting 34 interviews. However, we only interviewed 30 participants because three program sites did not hold delayed-intervention classes because of low attendance, and we could not reach a delayed-intervention participant from one of the sites.

#### Qualitative telephone interviews

All interviews were conducted by one of the two study interviewers (LS and BS) between November 2004 and April 2005. We developed a semistructured interview guide to examine whether participants thought ALED was an appropriate program for people with arthritis and to generate recommendations about how the program might be modified to better meet the needs of people with arthritis. We based our questions on key areas of interest: program safety, knowledge gained from the program, qualities of the instructor, and recommendations for modifying the program content for people with arthritis. Interviews were audio-recorded and lasted an average of 15 minutes (range: 7–29 minutes). The two study interviewers met frequently and reviewed all interview audiotapes to ensure that they administered the telephone interviews in a similar manner. The interview guide was revised as needed.

#### Instructor conference calls

We conducted two conference calls with a total of 14 ALED instructors to query them about their experience teaching the class. (Six instructors were unable to participate in either conference call because of scheduling conflicts.) The conference calls, which lasted about an hour, followed a structured format, were moderated by the study's principal investigator (LFC), and were audio-recorded.

### Data analysis 

Each interview with course participants was audio-recorded and transcribed verbatim. An independent researcher then listened to a random sample of the audiotapes to ensure that the transcripts were complete. We analyzed participants' responses using NUD*IST (N6) (QSR International, Melbourne, Australia), a software program for qualitative data analysis.

We developed an initial list of deductive codes based on the semi-structured interview questions. The study interviewers led the analysis and code development. Using the constant comparison method (25), they reread all transcripts each time a new theme emerged to ensure consistent coding. An independent coder (LFC) read through all of the transcripts, reviewed the initial coding scheme, and revised the scheme if necessary to increase the completeness of the findings. Members of the research team discussed discrepancies in coding until they reached a consensus. Common themes were identified on the basis of the frequency of responses across all interviews.

Immediately following each of the two conference calls with instructors, we summarized the main themes of the call. The calls were then transcribed verbatim and reviewed for the presence of other themes in the same manner as the transcripts of participant interviews.

## Results

Of the 30 participants interviewed, 27 (90%) were female, and their average age was 69 years (range: 50–83 years). Eighty percent were white, and 70% had at least some college education. Their average HAQ-DI score was 1.0, their average pain VAS score was 41, and their average body mass index (BMI; weight in kilograms divided by height in meters squared) was 31. Table 1 shows demographic characteristics for the full study sample, as well as for the subsample discussed here.

Our qualitative evaluation revealed that all participants felt safe in the program and thought it was appropriate for people with arthritis. All instructors also felt the program was appropriate for people with arthritis. No adverse events were reported during the course implementation. When queried about changes in their arthritis symptoms between the beginning and the end of the course, most participants reported that their symptoms improved, and the rest reported that their symptoms remained the same.

### Existing components of ALED that are particularly helpful to people with arthritis

Most participants reported finding two components of the ALED course particularly helpful to people with arthritis (Table 2). The first was being able to exercise at their own pace and "bit by bit" (e.g., 2-minute walks). The second was having social support from other adults with arthritis as they attempted to increase their level of physical activity. This social support included the opportunity to exchange information and ideas about pain management and living with chronic disease.

### Proposed modifications to make the ALED course more beneficial to people with arthritis 

Although the participants and the instructors both generally felt that the program was appropriate for people with arthritis, they did suggest some minor modifications. Instructors suggested modifications to the instructor training that would help them better prepare to teach the course to people with arthritis, whereas the participants generally suggested minor modifications not essential to the safety or appropriateness of ALED. Table 3 summarizes the proposed modifications and provides selected quotes from participants and instructors on three themes: instructors' training, handbook, and support; program content; and the ALED textbook.

#### Instructors' training, handbook, and support

Most ALED instructors did not have prior experience working with people with arthritis. Pain was the primary barrier to physical activity reported by ALED participants, and the instructors generally felt that the ALED goals and activities needed to be modified to address the need for pain management for people with arthritis. All instructors recommended that more information about arthritis and pain management strategies be provided to instructors, both during the ALED training session and throughout the course. They also recommended providing instructors with additional arthritis resources (e.g., handouts) and access to someone working at an arthritis program, such as a coordinator or director of a state arthritis program (Table 3).

The standard ALED course does not provide ongoing instructor support after instructors complete their training. As noted in the methods section, however, instructors in the RCT were provided with a toll-free number and a listserv to give them an opportunity to correspond with project directors and with each other. During the conference call, instructors emphasized the helpfulness of the toll-free number; however, few reported using the listserv.

#### Program content

A common theme in the comments of both instructors and participants was that the course could be made more relevant for people with arthritis by addressing how pain acts as a barrier to their engaging in physical activity. A significant component of the ALED program is to encourage participants to create goals that are attainable, given their lifestyle and current physical condition. Instructors were able to reinforce this need for attainable physical activity goals and elicited discussion among class participants about how their arthritis affected their participation in physical activity. Participants reported that because of the approach of the program and their interaction with their classmates, they were not afraid of hurting themselves or pushing themselves beyond a safe limit (Table 3).

Some participants said they appreciated a naturalistic approach to managing arthritis pain because they wanted to avoid taking more medication. Participants also generally reported that they liked discussing lifestyle factors beyond physical activity in the class, particularly factors such as diet and nutrition. Many indicated that they would have liked to learn more about weight management, the role of diet in managing arthritis symptoms, and how to identify healthy foods for people with arthritis.

#### ALED textbook

As noted above, each participant received an ALED textbook. When asked, most participants suggested modifications to the textbook to make it more appropriate for people with arthritis. The most frequently suggested modifications involved addressing pain management, exercise intensity, and the appropriateness of different types of exercise that target various forms of arthritis or pinpoint the most affected body parts. Some participants did not feel that the textbook needed to be modified and explained that the instructor did a good job of tailoring the class to meet the unique needs of people with arthritis. Others stated that while arthritis-specific modifications to the textbook may not be helpful to them, such changes may benefit those who have more severe arthritis-related problems or different forms of arthritis than they have.

### Influence of pain level and difficulty in ADL performance on participants' responses 

We were also interested in how participants' level of pain and level of ADL difficulty may have affected their recommendations for arthritis-specific modifications to the course. From the 30 participants in our qualitative analysis, we selected those with a baseline HAQ-DI score greater than or equal to 1.0 and a VAS pain score greater than 40 to represent people with moderate or higher amounts of arthritis-related disability and pain (26). Nine participants met these criteria. We found no obvious commonality among the responses of these participants. Some of them had had arthritis for many years and felt that they knew a lot about the disease and were already familiar with the arthritis-specific information discussed in class. Others who also had had the disease for many years were unfamiliar with the arthritis-specific information discussed in the course, such as what rheumatologists are and how exercise can be beneficial for people with arthritis. A few of these nine participants reported that their arthritis had become so severe that they had previously felt they could not exercise at all but that the ALED course showed them that they could be physically active again. All nine reported that they felt safe in the class because they trusted the instructor, knew their own limits, and learned that they could incorporate physical activity into their lives "bit by bit."

## Discussion

The results of our qualitative evaluation of ALED show that it is possible to take a program that was developed for a general population and implement it safely and appropriately in a population of people with arthritis. However, they also indicated that instructors without a background in working with people with arthritis would benefit from more specific information about arthritis and pain management strategies during training and from having access to arthritis brochures and an arthritis expert while teaching the course (as the RCT instructors did). Participants and instructors both generally felt that the course content could be improved by adding some arthritis-specific information (particularly regarding pain management) and by providing more relevant examples of physical activities appropriate for people with arthritis. Overall, though, the course as offered appeared to be robust and well received by participants with different types of arthritis and various levels of disease activity and to be a useful option for promoting physical activity among people with arthritis. Aspects of the course that were particularly appealing to people with arthritis, such as encouraging them to tailor their goals and activities to their own situation and to exercise "bit by bit," may also appeal to people with other chronic conditions.

This investigation may have been limited by participant recall because many of the interviews were conducted nearly 6 months after the participants completed the course. The time lapse sometimes made it hard for participants to remember details about their experiences in the class, particularly in regard to the course book, with as much candor as those interviewed soon after the class ended. Another limitation is that we interviewed only the participants who attended at least half of the classes offered, the completers. Therefore, we may be missing a valuable perspective from the noncompleters. However, from the attendance records kept by the course instructors, we do know that 43% of the noncompleters dropped out early in the course and never returned. These participants cited personal or family illness, scheduling conflicts, or the course not being a good match as primary reasons for dropping out of the program. The remaining noncompleters missed classes at various points throughout the entire course because of personal or family illness, scheduling conflicts (doctor appointments most frequently cited) and other outside factors. A final limitation to our findings is that they may not be generalizable to men, given that 90% of our participants were women. Women account for 60% of arthritis cases (1), so our participants are more heavily weighted for women, and it is possible that the program may need different or additional modifications for men with arthritis.

The lessons learned from our evaluation of ALED may be useful in modifying other physical activity programs designed for general populations and for use by people with arthritis, particularly in ensuring that they address the health issues that are most important to people with arthritis (Table 4). We recommend that anyone contemplating using an existing physical activity or behavior modification program that has not been developed specifically for people with arthritis consider the following modifications: 1) provide arthritis-specific education for instructors, with an emphasis on pain management; 2) provide instructors with arthritis-related resources and contacts with arthritis experts; 3) modify program material to include examples of physical activity appropriate for people with arthritis; 4) enhance program material with information on how to exercise while protecting arthritic joints and managing arthritis symptoms; and 5) incorporate information on dietary and other complementary strategies for alleviating arthritis symptoms. Our findings also indicate that physical activity classes for people with arthritis should encourage participants to exercise at their own pace, "bit by bit" if they prefer, and for instructors to create a class environment that fosters communication and friendship among class members. In conclusion, expanding the menu of community-based physical activity programs geared toward people with arthritis is a promising approach to increasing their health and well-being.

## Figures and Tables

**Table 1:** Demographic and Health Status Characteristics of Participants in a Randomized Control Trial of the Active Living Every Day (ALED) Program, North Carolina, 2004.

Characteristic	Qualitative Subsample (n = 30)	Full Group (n = 355)
Mean age in years (SD)	69 (10)	69 (10)
Female, %	90	84
White, %	80	78
>High school degree, %	70	60
Mean HAQ-DI score (SD)	1.0 (0.7)	0.9 (0.6)
Mean VAS pain scale score (SD)	41 (22)	42 (27)
Mean BMI (SD)	31 (7)	30 (7)

HAQ-DI indicates Health Assessment Questionnaire-Disability Index, which is scored on a scale of zero (indicating no difficulty in performing an activity) to 3 (indicating an inability to perform an activity); VAS, visual analogue scale, which is a scale of perceived pain from zero (indicating no pain) to 100 (indicating maximum possible pain); BMI indicates body mass index (weight in kilograms divided by height in meters squared).

**Table 2:** Existing Components of ALED That Participants Found Particularly Helpful for People with Arthritis, North Carolina, 2004

Component	Participants' comments	Rationale
Instructions to exercise bit by bit	"[These instructions]…encouraged us…[I]nstead of having to take a 30-minute walk, you could take it in spurts…do it in 5 or 10 minutes."	These instructions encourage participants to exercise in small spurts as a pain management technique.
Social support	"You didn't feel alone. You felt like whatever you presented to the class, someone knew what you were talking about." "…[I]t reminded me of Alcoholics Anonymous (laughing). [I]f you find yourself slipping or in a tight spot, you could call somebody and say, 'You know, I'm not doing what was said we should be doing.' It would give you a little encouragement…for somebody to say to you, 'Well, you have to do it slowly,' or 'Get back into the routine,' you know."	Instructors should create a class environment that fosters communication and friendship among class members. Instructors may suggest that class members call each other if they need encouragement to be physically active.

**Table 3:** Proposed Arthritis-Specific Modifications to the ALED Program, With Associated Comments by Participants and Instructors, North Carolina, 2004

Topic Area of Proposed Modification	Comments by Participants and Instructors	Proposed Modification
**Instructor training, handbook, and support**		
Arthritis	"A lot of people started asking…specifically why exercise is good for people with arthritis." (Instructor) "I didn't know a lot about arthritis, but all of the information that…[we received]…helped me understand a little bit more…[about the needs of]…people with arthritis." (Instructor)	Include a short section in the instructor handbook that discusses a basic overview of arthritis and the impact of physical activity on joints.
	"[For] people with arthritis, the number one reason [not to exercise] is pain. And I really didn't have an answer for what to do if you have too much pain….I don't want to tell…[class participants to push themselves if they]…have pain." (Instructor)	Include a short section in the instructor handbook that addresses pain management for people with arthritis. Instructors should have the information they need to appropriately tailor exercise goals and activities for the participants.
Access to arthritis resources	"It is extremely comfortable to have this partnership with the university…[T]hey had the 1-800 number…[that class participants] could call with…specific questions [about arthritis that]…we weren't equipped to answer…." (Instructor)	Provide handouts from the National Institute of Arthritis and Musculoskeletal Skin Diseases and the Arthritis Foundation to instructors during training and during the course. If possible, have a contact from a state public health agency available for questions by phone or e-mail.
**Program content**		
Pain management	"You know a lot of concerns were the pain. 'I can't do this.' But the techniques that were given in the book allowed…[course participants] to feel OK that if they had a lot of pain one week or one day, that they could reset their goals for the next week." (Instructor)	Instructors should emphasize concrete pain management techniques during the course, such as working at your own pace and exercising "bit by bit."
Information on diet, nutrition, and complementary strategies for arthritis management	"You always hear these advertisements about natural foods that are supposed to stop the pain. I would have liked to learn more…[about] that." (Participant)	Incorporate a lesson on diet and nutrition into the ALED program.
Arthritis-specific content in ALED textbook	"I think the book was very good, but it might have helped if…[it] had suggested different things for people with arthritis.…I think that some of the stuff in the book was a little too hard for us to do." (Participant) "A lot of people don't know too much about their body…. I think there needs to be some diagrams and something to show the knee and the hip and what…[they do]." (Participant)	Add a short section to the textbook that addresses pain as a barrier to physical activity. This section should also address how to protect against injury, monitor and manage symptoms, and accommodate exercise according to symptoms.

**Table 4:** Summary of Recommendations for Arthritis-Specific Modifications to Existing Community-Based Physical Activity Programs

Include arthritis-specific education for instructors, with emphasis on pain management.
Provide instructors with contacts and resources for arthritis information.
Modify program material to include examples of physical activity appropriate for people with arthritis.
Enhance program material with information on how people with arthritis can exercise safely while protecting their joints and managing their arthritis symptoms.
Incorporate information on diet, nutrition, and complementary strategies for arthritis management.
